# Production and characterization of monoclonal antibodies against *Encephalitozoon intestinalis* and *Encephalitozoon* sp*.* spores and their developmental stages

**DOI:** 10.1186/s13071-017-2503-z

**Published:** 2017-11-09

**Authors:** Fernando Izquierdo, Hercules Moura, Fernando Jorge Bornay-Llinares, Rama Sriram, Carolina Hurtado, Ángela Magnet, Soledad Fenoy, Govinda Visvesvara, Carmen del Aguila

**Affiliations:** 1 Laboratorio de Parasitología, Universidad San Pablo CEU, Madrid, Spain; 2 Centers for Disease Control and Prevention, Atlanta, GA USA; 3 Universidad Miguel Hernández de Elche, Elche, Spain

**Keywords:** *Encephalitozoon intestinalis*, *Encephalitozoon* sp., Monoclonal antibodies, Spores, Developmental stages, Diagnosis

## Abstract

**Background:**

Microsporidia are intracellular obligate parasites traditionally associated with immunosuppressed patients; their detection in immunocompetent patients has increased, highlighting their possible importance as emerging pathogens. Detection of spores in stools, urine, body fluids and tissues is difficult and immunological techniques such as immunofluorescence have proved to be a useful and reliable tool in the diagnosis of human microsporidiosis. For this reason, we have produced and characterized monoclonal antibodies (MAbs) specific for *Encephalitozoon intestinalis* (the second most frequent microsporidian infecting humans), and other *Encephalitozoon* species, that can be used in different diagnostic techniques.

**Results:**

Seven MAbs were selected in accordance with their optical density (OD). Four (4C4, 2C2, 2E5 and 2H2) were isotype IgG2a; two (3A5 and 3C9) isotype IgG3, and one Mab, 1D7, IgM isotype. The selected monoclonal antibody-secreting hybridomas were characterized by indirect immunofluorescence antibody test (IFAT), enzyme-linked immunosorbent assay (ELISA), Western blot, immunoelectron microscopy (Immunogold) and in vitro cultures. The study by IFAT showed different behavior depending on the MAbs studied. The MAbs 4C4, 2C2, 2E5 and 2H2 showed reactivity against epitopes in the wall of the spore (exospore and endospore) epitopes located in *Encephalitozoon* sp. spores, whereas the MAbs 3A5, 1D7 and 3C9 showed reactivity against internal epitopes (cytoplasmic contents or sporoplasm) of *E. intestinalis* spores. All MAbs recognized the developing parasites in the in vitro cultures of *E. intestinalis*. Additionally, 59 formalin-fixed stool samples that had been previously analyzed were screened, with 26 (44%) presenting microsporidian spores (18 samples with *E. intestinalis* and 8 samples with *Enterocytozoon bieneusi*). Detection of microsporidian spores by microscopy was performed using Calcofluor stain, Modified Trichrome, Quick-Hot Gram Chromotrope, as well as IFAT using MAbs 4C4, 2C2, 2E5 and 2H2. The 4 MAbs tested clearly recognized the larger spores corresponding to *E. intestinalis*, but showed no reactivity with *Enterocytozoon bieneusi* spores. The mass spectrometry and proteomic study revealed that the Mabs 4C4, 2C2, 2E5 and 2H2 recognized the Spore Wall Protein 1 (SWP1) as the antigenic target.

**Conclusions:**

The IFAT-positive MAbs exhibited excellent reactivity against spores and developmental stages, permitting their use in human and animal diagnosis. The epitopes recognized (exospore, endospore and cytoplasmic contents) by the different MAbs developed need further study, and may reveal potential targets for vaccine development, immunotherapy and chemotherapy.

## Background

Microsporidia are intracellular obligate parasites, ubiquitous in nature that can infect all animal phyla [1, 2]. Considered as human opportunistic pathogens, microsporidiosis is traditionally associated with immunosuppressed patients, such as those that are HIV-positive [3]. Nonetheless, their detection in immunocompetent patients has increased, highlighting their possible importance as emerging pathogens. However, the real prevalence of human infection has been underestimated [2, 4, 5], due to the difficulty of their diagnosis.

Detection of spores in stools, urine, body fluids and tissues is difficult, although the development of reliable differential staining techniques, such as Weber’s chromotrope-based stain and its modifications [6, 7], has improved the diagnosis of microsporidia. Nonetheless, these techniques are time consuming and intensive training is necessary to use them reliably. Moreover, diagnosis may be missed if the parasite burden is low or misdiagnosis can occur due to the presence of small yeast and/or sporulated bacteria in the samples that also stain. Morphological characteristics of the spores are ineffective in the identification of species, essential when choosing an appropriate treatment [8]. On the other hand, microsporidial DNA detection using molecular methods (mainly PCR) appears to be the most sensitive and specific method for species identification, but is expensive and not affordable in many clinical diagnostic laboratories. Furthermore, the presence of inhibitors of the polymerase enzyme in clinical samples (mainly stools), discourage their use [9–12].

In previous years, various studies have described the production and characterization of policlonal and monoclonal antibodies that recognize genus- and/or species-specific antigens of different microsporidia. Immunological techniques such as immunofluorescence has proved to be a useful and reliable tool in the diagnosis of human microsporidiosis [5, 12–27].

For this reason we have produced and characterized monoclonal antibodies (MAbs) specific for *Encephalitozoon intestinalis* (the second most frequent microsporidian infecting humans) and other *Encephalitozoon* species (*E. cuniculi* and *E. hellem*) that can be used in different diagnostic techniques.

## Methods

### Biological samples

#### Microsporidian spores

*Encephalitozoon intestinalis* (CDC:V297, provided by Dr Govinda Visvesvara, CDC, USA) [28], *Encephalitozoon cuniculi* (USP-A1, provided by Dr Carmen del Águila, USP-CEU, Spain) [29] and *Encephalitozoon hellem* (PV-5-95, provided by Dr Massimo Scaglia) [30] were cultured in Vero-E6 cells (provided by Dr Govinda Visvesvara, CDC, USA), harvested weekly, pooled, purified separately and stored at 4 °C until use, according to the method previously described by Visvesvara et al. [31]. *Enterocytozoon bieneusi* spores were obtained from an HIV-infected patient fecal sample provided by Dr Carmen del Águila (USP-CEU, Spain).

#### Microsporidian soluble antigen

Glass beads were used for disruption of the purified *Encephalitozoon* isolates in 2.5% SDS/10% 2-ME, using FP120 FastPrep™ Cell Disruptor (Bio 101, Cedex, France) and the protein content was determined by the Bradford method as described previously [32].

#### Enteropathogenic bacteria and other intestinal parasites

MAbs were assessed for cross-reactivity to enteropathogenic bacteria (*Proteus vulgaris*, *Pseudomonas aeruginosa*, *Escherichia coli*, *Shigella dysenteriae*, *Salmonella typhi*, *Yersinia enterocolitica*, *Enterococcus faecalis*, *Vibrio cholera*, *Klebsiella pneumonia* and *Enterobacter aerogenes*) provided by the Hospital Instituto de Salud Carlos III and the Microbiology Laboratory USP-CEU (Madrid, Spain), and the intestinal parasites (*Giardia* sp., *Entamoeba histolytica*, *Entamoeba coli*, *Cryptosporidium* sp., *Cyclospora* sp., *Isospora* sp. and *Blastocystis* sp.) provided by the Hospital Gregorio Marañón and Instituto de Salud Carlos III (Madrid, Spain).

#### Human fecal samples and ethics statement

Twenty-six human fecal samples obtained from HIV-positive and microsporidia-positive patients from Atlanta (CDC, Georgia, USA) were selected for this study. These samples had previously been identified by staining and molecular sequencing as *E. intestinalis*-positive (18 samples) and as *E. bieneusi*-positive (8 samples) [23]. These samples were stored at 4 °C and preserved in formalin. Use of trade names is for identification only and does not imply endorsement by the Centers for Disease Control and Prevention/ the Public Health Service, or the U.S. Department of Health and Human Services.

### Animals and immunization protocol

Hybridoma development and MAbs production was performed in adult (7-week-old) female BALB/c mice purchased from Charles Rives Laboratories. All mice were housed in filter-topped plastic cages with wood chip bedding and maintained at 12 h photoperiod cycles. Food and water were provided ad libitum.

Three immunization protocols were performed using three groups of six mice in each. All animals were immunized intraperitoneally (i.p.) with *E. intestinalis* (5 × 10^7^ spores per 200 μl). In protocols (immunizations) 1 and 2, non-purified and purified spores of *E. intestinalis*, respectively, were used in five inoculations (days 0, 7, 21, 35 and 42). In the third protocol (immunization), purified spores of *E. intestinalis* were emulsified at a 1:1 ratio in Freund’s adjuvant (Sigma-Aldrich Laboratories, Saint Louis, MO, USA) in four inoculations (days 0, 21, 42 and 56). For the first inoculation, Freund’s complete adjuvant was used; Freund’s incomplete adjuvant was used for subsequent inoculations.

In order to determine which immunization protocol produced the highest parasite specific antibody responses, mice sera were screened by indirect immunofluorescence antibody (IFAT). Sera were stored at -80 °C and used as positive controls.

### Production of MAbs

The MAbs were produced using the Köhler and Milstein method with modifications [33]. Three or four days before cell fusion, selected mice were boosted intravenously by an antigen inoculum (5 × 10^7^ spores in 50 to 100 μl of sterile saline solution). The spleen cells from the donor mouse were collected and fused with a non-secreting murine myeloma cell line, P3X63-Ag8.653 (BALB/c MOP21. ATCC#CRL-1580™) at a 5:1 ratio, using 50% polyethylene glycol (PEG). The cells were distributed into 96-well tissue culture plates with a feeder layer, at a concentration of 1–3 × 10^6^ cells/ml (100 μl/well). The hybridomas were selected by growth in a selective medium (RPMI-1640, 18% fetal bovine serum and 2 mM L-glutamine with hypoxanthine-aminopterin-thymidin (HAT) without antibiotics), and placed at 37 °C in 5% CO_2_. After 15 days, the undiluted culture supernatants were screened for the presence of anti-*E. intestinalis* antibodies by ELISA and IFAT. Hybridoma supernatants that showed antibody activity against *E. intestinalis* were expanded on to 24-well plates and cloned twice by limiting dilution.

### Enzyme-linked immunosorbent assay (ELISA)

ELISA 96 microtitter plates (Nunc-Immuno Plate PolySorp™, Darmstadt, Germany) were coated with *E. intestinalis* soluble antigen (0.8 μg/ml) as described previously [4]. Hybridomas with an optical density (OD) greater or equal to 0.5 were selected following the criteria of Pomport-Castillon et al. [34].

### Immunofluorescence antibody test (IFAT)

#### Hybridoma screening

*E. intestinalis* purified spores were used to screen hybridoma supernatans and to determine the specificities of MAbs. The procedure was performed as described previously [28].

#### Development of *E. intestinalis* in cell culture and immunolabeling of different stages

Vero-E6 cells were cultivated in culture chambers with 2 wells (4.2 area cm^2^/well, Ref. 155,380, Lab-Tek®) and infected with purified spores (2 × 10^5^ spores/ml). The developmental stages were monitored by double labeling using fluorescein and rhodamine at 24, 48 and 72 h post-infection. A hyperimmune polyclonal rabbit sera [32] that recognizes all development stages of *E. intestinalis* was labeled with fluorescein and the MAbs (undiluted) labeled with rhodamine were used in order to determine the epitopes recognized by the MAb.

#### Cross-reactivity studies

MAbs were also assessed by IFAT for their cross-reactivity to other microsporidia (*E. cuniculi*, *E. hellem* and *E. bieneusi*), enteropathogenic bacteria, and other intestinal parasites as described previously.

#### Diagnosis of microsporidiosis in human fecal samples

Thin smears were prepared from each of the unconcentrated human fecal samples and tested for the presence of microsporidian spores by Calcofluor (CF) [17], Modified Trichrome stain (MT) [6] and Gram-chromotrope stain (GC) [35]. IFAT with MAbs (4C4, 2C2, 2E5 and 2H2) was performed on the fecal samples using fluorescein Isothiocyanate-conjugated anti-mouse (Sigma Cat. F-1010, Saint Louis, MO, USA). Finally, Calcofluor and IFAT were performed on the same samples to compare the sensitivity and specificity between these fluorescent techniques.

### Isotyping

MAb isotype and light chain composition were determined by isotyping kit Dipstick Format according to the manufacturer’s instructions (Gibco-ThermoFisher, Waltham, MA, USA).

### Western blot

SDS-polyacrylamide gel electrophoresis (SDS-PAGE) was used to determine soluble protein antigen profiles of *E. intestinalis*, *E. cuniculi* and *E. hellem* [29].

### Immunoelectron microscopy

*Encephalitozoon intestinalis* spores were fixed with 2% paraformaldehyde/ 0.2% glutaraldehyde in 0.1 M cacodylate buffer (pH 7.4). After 1 h the spore suspension was centrifuged at 2100× *g* for 30 min and the pellet washed in 0.1 M Na-cacodylate buffer and 4.5% sacarose. After dehydration with ethanol, the material was embedded in Lowicryl K4 M resin. The sections were collected on nickel grids. Spore sections were blocked with 1% BSA in TBS for 1 h at 37 °C and later incubated for 1 h with the monoclonal antibody (1:1, 1:2, 1:5 and 1:10 dilutions of each). After intensive washings in BSA (0.1%)-TBS (Tris-buffered saline, pH 7.4), sections were incubated for 1 h at 37 °C with goat anti-mouse IgG/IgM labelled with 5 and 10 nm gold particles (Auroprobe GAM, Amersham). Controls consisted of sections incubated with the secondary antibody alone. After washing, the grids were stained with 1% uranyl acetate for 10 min before examination in a transmission electron microscope.

### Mass spectrometry and proteins

Proteomic methods were used to determine the possible epitopes recognized by the four IFAT-positive MAbs (4C4, 2C2, 2H2 and 2E5). For this study, the species selected was *E. cuniculi* (USP-A1) due to the good profiles and protein fractions recognized by all MAbs using Western blot. One set of gels was transferred to blot membranes in order to determine the immunoreactivity profiles of the MAbs (dilution 1:20, iBlot™ Dry Blotting System, Invitrogen™ Carlsbard, CA, USA). This assay permits the identification of the separated proteins recognized by the MAbs. SDS-PAGE analysis (GeLCMS) and in-gel digestion were performed as described previously [36]. Briefly, NuPage® Novex® 4–12% Bis-Tris Mini Gels (Invitrogen™, Carlsbard, CA, USA) were used to determine soluble protein profiles of *E. cuniculi* stained with Coomasie Blue. Each gel lane was cut into 20 slices, dehydrated and digested with sequencing grade modified trypsin (Promega™, Madison, WI, USA) and then incubated for 16 h at 37 °C. The digests were quenched with 0.2% formic acid, sonicated and centrifuged. The supernatants were used for nanoscale high-pressure liquid chromatography (nHPLC-MS/MS) analysis.

Tryptic digests were analyzed by MS. nLCMS/MS was carried out using a nanoAcquity UPLC coupled to a QTOF Premier MS system (Waters Corporation, Milford, MA, USA). nLC separation was performed using a Symmetry C18 trapping column and a BEH C18 column (100 μm ID × 100 mm long with 1.7 μm packing), at a flow rate of 1.2 μl/min, at 35 °C. Standard water/ACN gradients containing 0.1% formic acid in both solvents were used for elution, with the ACN increasing linearly from 1% to 50% in 50 min, and an overall cycle time of 90 min. The QTOF used an MS^E^ (or Protein Expression) method, which involved acquiring data-independent alternating low- and high-energy (4 and 15–45 eV collision energy, respectively) scans over the m/z range 50–1990 in 0.6 s, along with lock mass data on a separate channel. The lock mass consisted of 100 fmol/μ^l^ of [Glu-1]-fibrinopeptide B and was delivered from the auxiliary pump with a constant flow rate of 300 nl/min. Quality control of the nLC-MS/MS runs was assured by adding to each queue a protein standard consisting of a tryptic digest of yeast ADH (Waters Corporation, Milford, MA, USA) before the first sample injection and again following the last sample injection. Each digest was analyzed in triplicate (three technical replicates per sample) and their respective raw data file obtained using the MS^E^ data-independent method was further processed using the PROTEINLYNX GLOBAL SERVER v2.4 software (PLGS, Waters Corporation), for protein identification and quantification. Database searches were performed using the PLGS Identity^E^ database search algorithm against a UniProt protein database and provided statistically validated peptide and protein identification along with relative and absolute protein quantification analysis. Protein identifications were based on the detection of a minimum of two peptides identified per protein and a minimum of seven total product ion matches per protein. The maximum false-positive rate against the randomized forward database was set to 1%.

All protein identifications were manually verified. The relative protein quantification was obtained using both the PLGS Identity^E^ and the Expression software in the samples spiked with a known amount of standard ADH digest (100 fmol on column). The clustered data set was exported from PLGS and further evaluated with Excel (Microsoft Corporation, Redmond, WA, USA). SCAFFOLD (v3.01, Proteome Software Inc., Portland, OR, USA) was used to further validate MS/MS-based peptide and protein identifications. Peptide identifications were accepted if they could be established at > 95.0% probability as specified by the Peptide Prophet algorithm. Protein identifications were accepted if they could be established at > 99.0% probability and contained at least two identified peptides.

## Results

### Production of MAbs

After nine fusions of *E. intestinalis*-immunized murine spleen cells with P3X63-Ag8.653 myeloma cells, seven antibody-secreting hybridomas showed reactivity against *E. intestinalis* were selected (Table 1). ELISA screened and selected the positive hybridomas by the optical density values obtained (OD) (Table 1). The studies by IFAT revealed that four of the seven MAbs (4C4, 2C2, 2E5 and 2H2) exhibited reactivity against the exospores of *Encephalitozoon intestinalis*, *E. cuniculi* and *E. hellem* (Table 1).

**Table 1:** Reactivity and characterization of MAbs to *E. intestinalis*

MAb	Immunization protocol	Isotype	ELISA (OD)	IFAT^a^	WB (KDa)^b^		EM	Specificity of the MAbs (use dilution by IFAT)	
					*E. intestinalis*	*E. cuniculi / E. hellem*		Genus	*E. intestinalis*
4C4	1	IgG_2a_	+ (1.482)	+	201–36.4 (45)^c^	205–36.8 (63 & 59.2)^c^	EXP/ENP	1:25–1:50	1:400^d^
2C2	3	IgG_2a_	+ (0.940)	+	201–36.4 (45)^c^	205–36.8 (63 & 59.2)^c^	EXP/ENP	Not diluted	≥ 1:50^d^
2H2	3	IgG_2a_	+ (0.859)	+	201–36.4 (45)^c^	205–36.8 (63 & 59.2)^c^	EXP/ENP	Not diluted	≥ 1:50^d^
2E5	3	IgG_2a_	+ (0.732)	+	48.3–36.4 (45)^c^	205–36.8 (63 & 59.2)^c^	EXP/ENP	Not diluted (negative for *E. hellem*)	≥ 1:25^d^
3A5	2	IgG_3_	+ (1.130)	–	16	–	CTPC	–	–
3C9	2	IgG_3_	+ (1.523)	–	40 & 16	–	CTPC	–	–
1D7	2	IgM	+ (0.756)	–	40 & 16	–	CTPC	–	–

^a^Mature spores

^b^Soluble antigen

^c^Well-defined band

^d^Without cross-reactions

*Abbreviations*: *MAb* monoclonal antibody, *+* positive reactivity, *−* no reactivity, *OD* optical density, *EM* electron microscopy, *EXP* exospore, *ENP* endospore, *CTPC* cytoplasmic contents

### Characterization of monoclonal antibodies

#### Isotyping

The isotyping of antibodies showed that four were IgG2a (4C4, 2C2, 2E5 and 2H2), two IgG3 (3A5 and 3C9) and one IgM (1D7) (Table 1).

#### Western blot

**Fig. 1:**
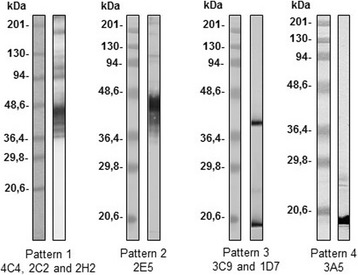
Western blot of SDS-PAGE (12%) separated proteins of *E. intestinalis*. Undiluted monoclonal antibodies: Pattern 1 (MAbs 4C4, 2C2 and 2H2); Pattern 2 (MAb 2E5); Pattern 3 (MAbs 3C9 and 1D7); Pattern 4 (MAb 3A5)

The study by Western blot using *E. intestinalis* soluble antigen determined four different patterns (Fig. 1), bands from 36.4 to 201 KDa (MAbs 4C4, 2C2 and 2H2) (Table 1, Fig. 1), 36.4 to 48.3 KDa (MAb 2E5) (Table 1, Fig. 1), all showing a well-defined band at 45 KDa. The third pattern showed a band of 16 KDa (MAb 3A5) (Table 1, Fig. 1) and the fourth, two bands around 16 and 40 KDa (MAbs 1D7 and 3C9) (Table 1, Fig. 1). With respect to *E. cuniculi* and *E. hellem* the MAbs 4C4, 2C2, 2H2 and 2E5 showed cross-reactions with bands from 36.8 to 205 KDa and two well-defined bands (63 and 59.2 KDa). Finally, MAbs 1D7, 3C9 and 3A5 showed an absence of reactivity to *E. cuniculi* and *E. hellem* (Table 1).

#### Cross-reactivity studies

The presence of possible cross-reactions with other microsporidia (*E. cuniculi*, *E. hellem* and *Enterocytozoon bieneusi*), and other enteric parasites and enteric bacteria were studied. None of the IFAT-positive MAbs exhibited cross-reaction with the enteric parasites and enteric bacteria studied. However, in the case of microsporidia, the four MAbs showed cross-reactions with *E. cuniculi* and *E. hellem* by IFAT and by Western blot. MAb 2E5 could be useful in the detection of *E. intestinalis* according to the dilution used (Table 1), due to the absence of cross-reactions and the four MAbs for *Encephalitozoon* spp. detection (undiluted saturated supernatant). In the case of *E. bieneusi*, no cross-reactions were detected.

### Immunoelectron microscopy

**Fig. 2:**
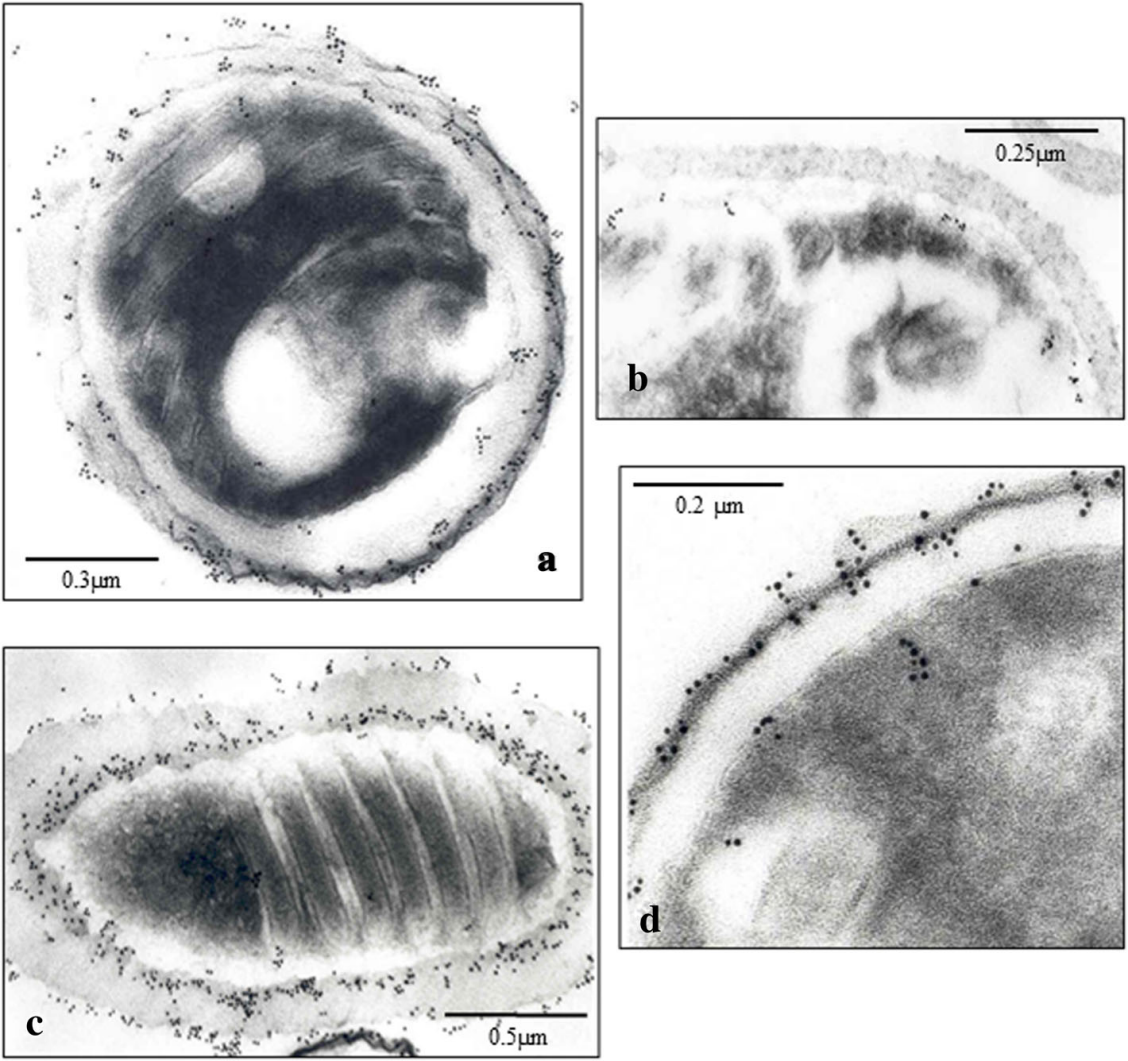
Immunogold electron micrographs of *Encephalitozoon intestinalis* spores after incubation with MAbs. Undiluted supernatant were used: 2C2 (**a**), 3C9 (**b**), 2E5 (**c**) and 4C4 (**d**). *Scale-bars*: **a**, 0.3 μm; **b**, 0.25 μm; **c**, 0.5 μm; **d**, 0.2 μm

The immunochemical characterization by electron microscopy (Immunogold) techniques showed that 3A5, 1D7 and 3C9 MAbs recognized internal epitopes of *E. intestinalis* spores (cytoplasmic contents) (Fig. 2b, MAb 3C9). However, the four IFAT-positive MAbs (4C4, 2C2, 2E5 and 2H2) reacted against epitopes of the wall of the spore (exospore and endospore) (Fig. 2a-d).

### Immunorecognized stage of development of *E. intestinalis* in cell culture

**Fig. 3:**
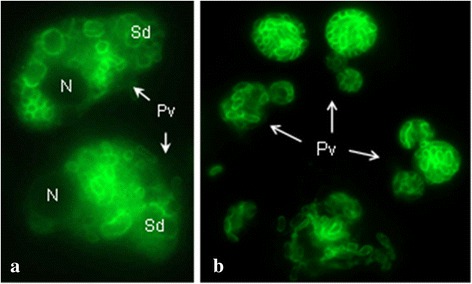
Immunorecognized in culture cell (Vero-E6) of *E. intestinalis* after incubation with MAb 2E5 (undiluted supernatant). **a** 24 hpi. **b** 72 hpi. *Abbreviations*: N, nucleus; Pv, parasitophorous vacuole; Sd, stages of development; hpi, hours post-infection

The cellular cultures infected with *E. intestinalis* spores were studied in order to describe the reactivity of the MAbs to the different stages of development of the spore. The seven MAbs showed recognition of developed parasitophorous vacuoles from the first hours of infection to 72 h. The developmental stages were also observed (Fig. 3). MAbs (4C4, 2C2, 2E5 and 2H2) and the polyclonal serum recognized perfectly the infectant mature spores.

MAbs (3A5, 1D7 and 3C9) only presented activity against the parasitophorous vacuoles detecting the stages of development that are inside. These same MAbs did not recognize or detect the infectant mature spores. Finally, none of the seven MAbs, recognized the extrude filaments.

### Diagnosis of microsporidian spores in human fecal samples by IFAT and staining methods

**Fig. 4:**
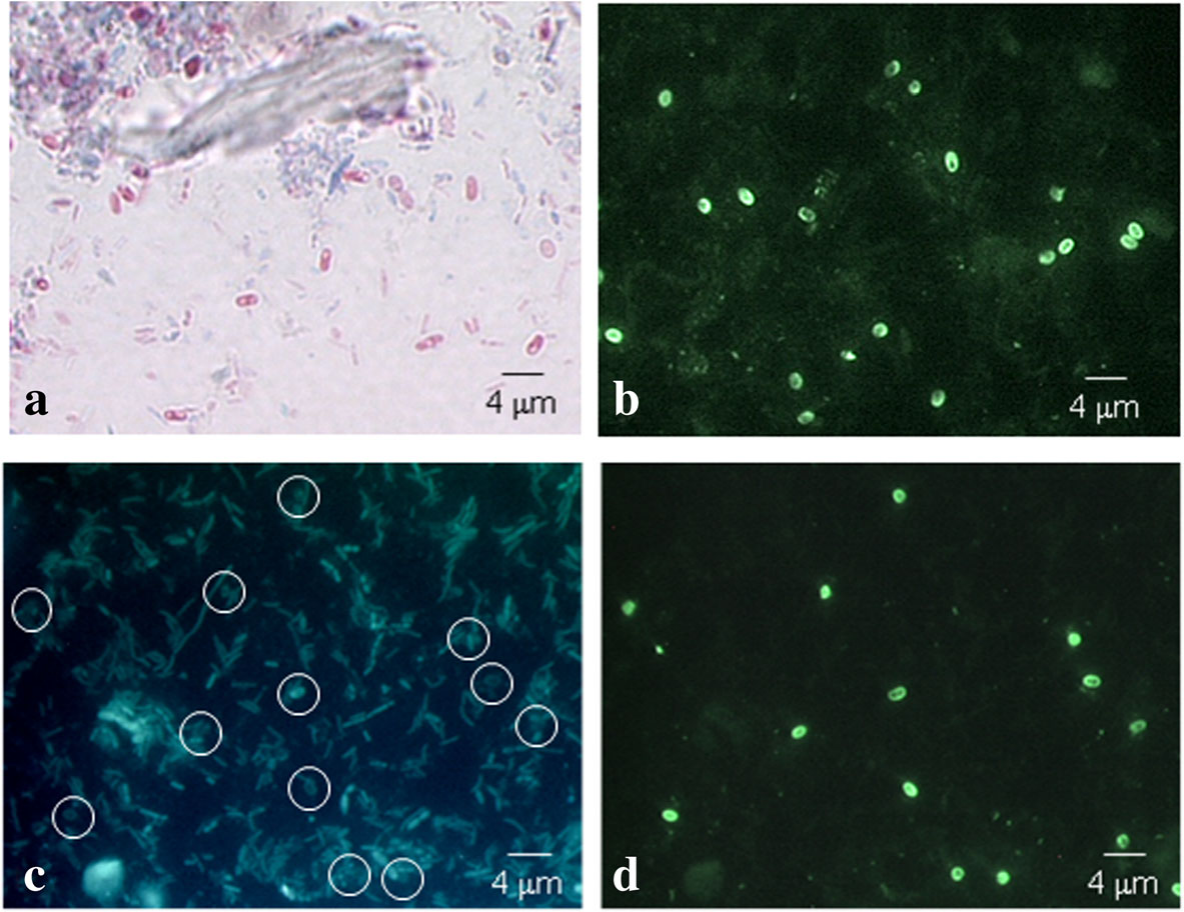
Formalin-fixed fecal samples from patients with microsporidia. **a** Fecal smear stained by the Modified Trichrome technique. **b** *Encephalitozoon intestinalis* spores detected by IFAT with the MAb 2E5. **c** and **d** are the same field: **c** Fecal smear stained by the Calcofluor technique. **d** Spores detected by IFAT with MAb 2C2. *Scale-bars*: 4 μm

The results obtained using IFAT with the MAbs agreed with those of the staining techniques. *Encephalitozoon intestinalis*-positive samples showed a positive reaction with the four MAbs that recognized the exospore while negative results were obtained when *E. bieneusi*-positive samples were tested (Table 2, Fig. 4a, b). However, the number of spores stained with CF was lower compared with the total number of spores observed in the other stains and by IFAT (Fig. 4c, d).

**Table 2:** Results of three staining techniques and IFAT for identification of microsporidian spores in fecal specimens from AIDS patients

Fecal positive samples (*n* = 26)		Staining methods			IFAT			
Species *n* (%)	Spore quantity	CF	MT	GC	MAb			
					4C4	2C2	2E5	2H2
*E. intestinalis* 18 (70%)	VN: 6	6^a^	6	6	6	6	6	6
	N: 1	1^a^	1	1	1	1	1	1
	F: 11	11^a^	11	11	11	11	11	11
*E. bieneusi* 8 (30%)	F: 8	8^a^	8	8	–	–	–	–

^a^Fewer spores per field

*Abbreviations*: *VN* very numerous, *N* numerous, *F* few, CF Calcofluor, *MT* Modified Trichrome, *GC* Gram-chromotrope

### Mass spectrometry and proteins

**Fig. 5:**
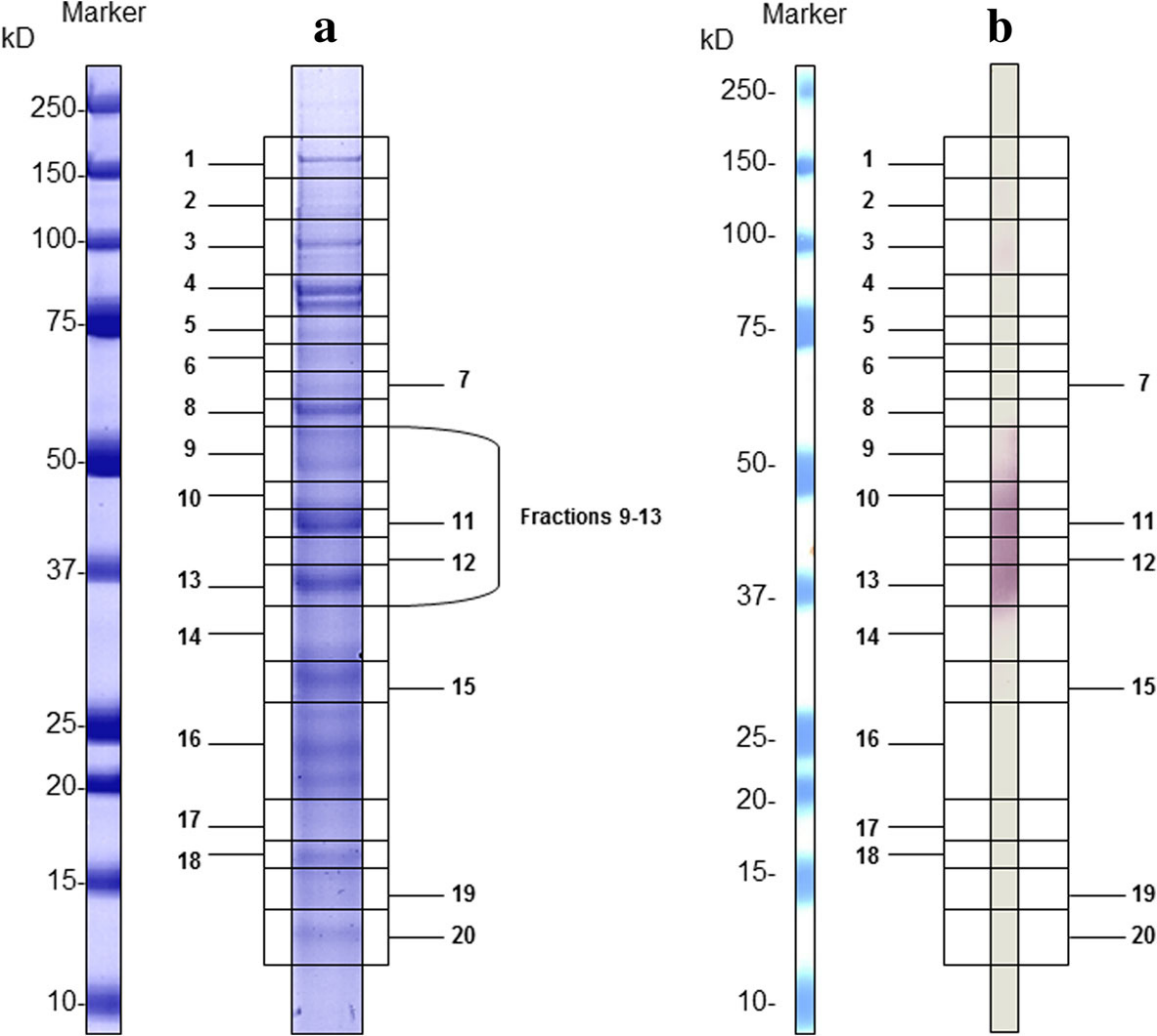
Soluble antigen profiles of *E. cuniculi.* **a** Soluble antigen in NuPage® gel stained with Coomasie Blue. **b** Immunoreactivity profile of MAb 2C2 (dilution 1:20, iBlot™ Dry Blotting System, Invitrogen™)

The four IFAT-positive MAbs (4C4, 2C2, 2H2 and 2E5) displayed the same profiles of immunoreactivity (iBlot™ Dry Blotting System) using soluble antigen of *E. cuniculi* (Fig. 5). The immunoreactivity profile allowed the clear identification of 5 fractions (9–13, Fig. 5) in a range between 37 to 50 KDa, with two well-defined bands, 37 and 45 KDa, in the fractions 13 and 11, respectively. Spectra of microsporidian protein extracts revealed the presence of different proteins in the 5 fractions studied (Table 3). It is important to highlight the detection of the Spore Wall Protein 1 (SWP1) only in the fractions 11 and 13, with a mass weight of 45,844 Da (Table 3). Moreover, in all fractions, other peptides corresponding to cytosolic proteins or cytoplasmic contents were identified.

**Table 3:** Proteins identified in the five fractions studied by Q-TOF MS/MS

No. of fractions	Accession number	Description	Protein mW (Da)
9	Q8SWI4_ENCCU	Putative protein ECU01 1270	22,492
	Q8SQL1_ENCCU	Protein disulfide isomerase	50,357
	Q8SQZ7_ENCCU	Cytosol aminopeptidase-LEUxPRO	52,355
	IF4A_ENCCU	ATP dependent RNA helicase	48,460
	Q8SU65_ENCCU	Similarity chito-oligosaccharide deacetylase	28,059
10	ACT_ENCCU	Actin *E. cuniculi*	41,966
	Q8SVP6_ENCCU	Putative protein ECU04 1630	49,997
	ABC1_ENCCU	Probable ABC transporter ECU01	65,515
	Q8SV25_ENCCU	Putative protein ECU07 0530	48,113
11	ACT_ENCCU	Actin *E. cuniculi*	41,966
	Q8SWL3_ENCCU	Putative protein ECU01 0820	40,557
	Q8SVN5_ENCCU	Putative protein ECU05 0110	36,091
	SWP1_ENCCU	Spore wall protein 1	45,844
12	Q8SQW6_ENCCU	U5 associated snRNP	36,571
	Q8SVT5_ENCCU	Putative protein ECU04 0990	25,815
	Q8SRB0_ENCCU	Guanine nucleotide binding protein beta subunit.	36,727
13	Q8SUE5_ENCCU	Putative protein ECU10 0880	35,379
	Q8SRT0_ENCCU	Polar tube protein PTP2	30,056
	ACT_ENCCU	Actin *E. cuniculi*	41,966
	Q8SR69_ENCCU	Inorganic pyrophosphatase	31,851
	Q8SSK6_ENCCU	Aldose reductase	33,335
	Q8SVN2_ENCCU	Putative protein ECU05 0140	51,285
	Q8SSD6_ENCCU	CAAX prenyl protease 1	47,329
	SWP1_ENCCU	Spore wall protein 1	45,844
	Q8SWI4_ENCCU	Putative protein ECU01 1270	22,492

## Discussion

The present study reports the production, characterization and reactivity of seven MAbs directed against *E. intestinalis*, the second most common microsporidia infecting immunodeficient patients. Microsporidian infections are difficult to diagnose because these parasites are difficult to distinguish from bacteria and small yeasts, mainly in stool samples. Staining methods such as Modified Trichrome (MT), Gram-Chromotrope (GC) and Calcofluor (CF), have been used in previous studies to detect microsporidia in clinical samples, but have shown some difficulties in interpretation [6, 17, 21, 37].

An immunofluorescence assay with monoclonal antibodies is a highly sensitive and specific technique, with lower background noise and without cross-reactivity with other bacteria, fungi or parasites, compared to polyclonal antibodies. Immunofluorescence assays using polyclonal serum produced in rabbits against *E. hellem*, *E. cuniculi*, *E. intestinalis* and *E. bieneusi* have shown the existence of cross-reactions among the different species of *Encephalitozoon* and with *E. bieneusi*, indicating the presence of common antigens between genus and species [38]. In addition, cross-reactions with bacteria and fungi have been observed, although morphological characteristics and size can help to distinguish them from the microsporidian spores [28, 39–44].

Seven MAbs were initially selected according to their optical density (OD). Four (4C4, 2C2, 2E5 and 2H2) were isotype IgG_2a_; two (3A5 and 3C9) isotype IgG_3_, and one MAb, 1D7, IgM isotype. The study by IFAT showed different behavior depending on the MAbs studied. The MAbs 4C4, 2C2, 2E5 and 2H2 showed reactivity against the exospore, whereas the MAbs 3A5, 1D7 and 3C9 were characterized by the absence of reactivity.

The study of cross-reactions by IFAT with other microsporidian species showed a significant recognition of the spores of *E. cuniculi* with respect to the spores of *E. hellem* for MAbs 4C4, 2C2, 2E5 and 2H2, when the undiluted supernatants were used. These results demonstrate the presence or absence of certain epitopes in each species and the possible presence of common genus epitopes [45, 46]. It is important to note that MAb 2E5 could be useful in the specific diagnosis of *E. intestinalis*. In the case of *E. bieneusi*, no cross-reactions were detected.

The results obtained by Western blot with soluble fractions of *E. intestinalis* confirmed the results by IFAT. MAbs 4C4, 2C2 and 2H2 recognized protein fractions from 201 to 36.4 KDa, as previously described by other authors [45–53], as well as an antigenic fraction of 45 KDa, recognized with significant intensity [53]. MAb 2E5 showed a recognition of more reduced bands within the range 48.3 to 36.4 KDa [51–53], and as in the three previous cases, the band recognized with significant intensity was 45 KDa [53].

Several studies have described and associated different bands characterized by Western blot with possible proteins such us the antigen targets recognized by MAbs [49, 50]. The four IFAT-positive MAbs (Pattern 1: 4C4, 2C2, 2H2 and Pattern 2: 2E5) all showed a well-defined band at 45 KDa. These results could relate to the protein EiSWP1 (≈50 KDa) which is localized in the exospores of mature spores [49]. The results obtained with these 4 MAbs in the cellular cultures infected with *E. intestinalis*, showed reactivity to the different stages of development of the spores in the parasitophorous vacuoles. This recognition will be again associated with the protein EiSWP1 located in the transition from the stages of meront to sporont and in the final stages of maturation (mature spores) [49]. These results might justify the minor presence of cross-reactions of the 2E5 antibody with respect to antibodies 4C4, 2C2 and 2H2 that recognize a large number of common epitopes among the *Encephalitozoon* species.

The results obtained by mass spectrometry and proteomics confirmed that the main epitope recognized by the IFAT-positive Mabs is SWP1, with a molecular weight (MW) of 45,844 Da (using soluble antigen of *E. cuniculi*). This result supports the results obtained by the MS result, which strongly confirms our MET findings which indicated the precise recognition of the exospore (spore wall) and total absence of reactivity against internal epitopes (cytoplasmic contents or cytosolic proteins). Although the MW obtained by Western blot [29] with these same Mabs against *E. cuniculi* were slightly different to the two well-defined bands of 63 and 59.2 KDa, these differences could be due mainly to the special and specific conditions of the study by proteomics such as: types and characteristics of gels used, treatment of the soluble antigen, conditions of electrophoresis and the separation of the peptides by LC (monomers, dimers or polymers of a same protein).

It is important to emphasize the total absence of reactivity of the four antibodies to *E. bieneusi* spores and, the absence of cross-reactions when the study was performed by IFAT with saturated supernatants against other enteric parasites and enteric bacteria [8, 46, 54]. These results support the use of the MAbs described in fecal samples for the diagnosis and/or confirmation of microsporidiosis produced by *Encephalitozoon* species.

MAbs 3A5, 1D7 and 3C9, with absence of reactivity in IFAT, exhibited different behavior with respect to the previous four. MAbs 1D7 and 9C9 recognized antigenic fractions of 40 and 16 KDa (Pattern 3) in the soluble antigen of *E. intestinalis* [51, 53] while the antibody 3A5 recognized only one band of 16 KDa (Pattern 4). It is important to highlight that they did not react with any antigens of *E. hellem* and *E. cuniculi*.

The protein of molecular weight 40 KDa is related with the sporogony, but the epitope recognized by the MAb becomes inaccessible showing a negative reactivity by IFAT [50]. However, the protein of 16 KDa could be compared with a band of ≈23 KDa described and associated with the sporogony phase [50]. The presence of several or multiple proteins (bands) recognized by the MAbs studied could be due to the presence of the epitopes present in different antigens or proteins, or in the same protein depending on the chemical treatment for the analysis by Western blot, or the presence of monomers, dimers or polymers of the same protein.

The results obtained by electron microscopy (Immunogold) confirmed the IFAT results obtained. 4C4, 2C2, 2E5 and 2H2 MAbs recognized the exospore, and the total absence of reactivity by IFAT of MAbs 3A5, 1D7 and 3C9 was due to their union with internal structures of the spore [13, 45, 50–52, 55].

Finally, seven MAbs were tested against infected Vero-E6 cells with *E. intestinalis* at 24, 48 and 72 h post-infection by IFAT with the aim of elucidating the antigenic distribution of the epitopes and their expression during the developmental stages. Three different patterns of immunoreactivity were established.

Included in the first pattern are the IFAT-positive MAbs (4C4, 2C2, 2E5 and 2H2). All of these recognized mature spores and the intraparasitophorous vacuole stages. These results suggest that the final components of the spore wall begin to be expressed in very immature stages of the parasite. In the second pattern, the antibodies 1D7 and 3C9 recognized exclusively developmental stages. These two MAbs were not able to recognize mature spores, suggesting the possibility that the epitopes are synthesized and expressed in the first phases of the development, the antigens remaining within in the cytoplasmic contents (sporoplasm) as cover antigens, undetectable in the mature stages of the spore by IFAT. This fact correlates with the results obtained in the studies by electron microscopy described above [13, 45, 50–52].

The third pattern of fluorescence was exhibited by MAb 3A5, which presented reactivity during all the developmental stages. The recognized epitopes appeared with a diffuse distribution inside of the parasitophorous vacuoles. Therefore, the recognized antigen would be located within the cytoplasmic contents, appearing not to be associated or related to the structural components of the parasite. Again, this behavior correlated with the results obtained by electron microscopy, confirming that the recognized epitope was located in the cytoplasmic contents, not associated with the wall of the parasite.

In order to establish the usefulness of MAbs in microsporida diagnosis, a comparative analysis using a reference technique (CF) was perfomred. The results obtained by IFAT with MAbs 4C4, 2C2, 2E5 and 2H2 appeared to be highly specific. *Encephalitozoon intestinalis* spores were identified in 18 positive fecal samples corresponding with the results of the staining techniques. The spores were easily distinguished with bright fluorescence by IFAT, while with CF in the same field the number of fluorescent spores was low, weak or absent, due to a possible degeneration of the chitin [17]. This could give rise to the presence of false negatives or the underestimation of the parasitic load. Moreover, sometimes CF could favor some false positive diagnosis due to the similarity in the staining of small yeast cells.

It is well-known that diagnosis of microsporidiosis at the species level is important for the treatment of patients. Molecular techniques present high sensitivity and specificity but may also present several disadvantages, such as being more expensive, time-consuming, the possible presence of inhibitors in the clinical samples and the need for qualified professionals [9–12]. The application of MAbs (4C4, 2C2, 2E5 and 2H2) in stool samples by IFAT showed a good sensitivity and specificity and allowed us to identify the microsporidian spores faster and easier than with CF, GC and MT, due to bright fluorescence of the spore wall that facilitates the diagnosis even to the untrained eye. These results indicate that *E. intestinalis* and *Encephalitozoon* spores can be identified in fresh and fixed stool samples and stored at 4 °C for several years.

## Conclusions

In summary, the different patterns of fluorescence observed in mature spores and in different stages of development suggest the possible utility of these 7 MAbs in the diagnosis of *E. intestinalis* and *Encephalitozoon* spp. in fecal and histological samples from human and animal hosts. This is the first report on the characterization and use of MAbs (4C4, 2C2, 2E5 and 2H2) in in vitro cultures that could permit their use in tissue samples or biopsies for the detection of the developmental stages. It can be concluded that the use of these MAbs could help in the study of the expression, distribution and characterization of epitopes, such as the SWP1 recognized by the Mabs (4C4, 2C2, 2E5 and 2H2), and be useful in the search of new therapeutic targets.

## Abbreviations

CF: Calcofluor stain
ELISA: Enzyme-linked immunosorbent assay
GC: Quick-Hot Gram Chromotrope stain
IFAT: Indirect immunofluorescence antibody test
MAb: Monoclonal antibody
MT: Modified Trichrome stain
OD: Optical density
